# COVID-19, medical education and the impact on the future psychiatric workforce

**DOI:** 10.1192/bjb.2020.112

**Published:** 2020-10-21

**Authors:** Flora Greig

**Affiliations:** South London and Maudsley NHS Foundation Trust, UK

**Keywords:** COVID-19, foundation programme, recruitment, education and training, medical students

## Abstract

Since the start of the COVID-19 pandemic the UK's National Health Service (NHS) has been transformed to meet the acute healthcare needs of infected patients. This has significantly affected medical education, both undergraduate and postgraduate, with potential long-term implications for psychiatric recruitment. This article discusses these ramifications, and the opportunities available to mitigate them as well as to enhance the profile of psychiatry.

When it became clear that the first two cases of SARS-CoV-2 infection reported in the UK in January 2020^1^ represented an emerging pandemic, the National Health Service (NHS) completely reconfigured its services. To meet the needs of patients diagnosed with COVID-19, elective treatments were cancelled, face-to-face appointments moved online and huge numbers of healthcare workers were redeployed. While mass transfers of staff to front-line roles were facilitated, movements of other healthcare workers were delayed or cancelled: 20 000 junior doctors due to rotate training placements on 1 April 2020 were informed by Health Education England that, except in exceptional circumstances, their rotations were cancelled.^2^ At the same time, medical students throughout the country faced widespread disruption of their studies.^3^ Both these events have resulted in a significant number of undergraduate and postgraduate medical trainees missing out on psychiatry placements, with potentially significant consequences for future recruitment into the specialty. These have not gone unnoticed.^4^

## The recruitment crisis in psychiatry and its wider workforce implications

Concerns about recruitment into psychiatry are not new. A review of medical student career choices over 40 years demonstrated that the number choosing to specialise in psychiatry has remained constant – at 4–5% – despite the increasing mental health needs of the population.^5^ Surveys of career choices of foundation doctors show a similarly fixed number applying for core psychiatry training.^6^ A recent estimate suggests that, if the projected increase in Certificate of Completion of Training (CCT) holders is accurate (based on numbers applying to training), by 2033 there will be a per capita reduction in psychiatrists of around 11%.^7^

A number of reasons for this crisis have been suggested. These include stigma towards the profession, perceived lack of a scientific basis to diagnosis and treatment, and erosion of the role of the psychiatrist within mental health teams.^8^ Poor teaching and clinical experience at medical school have also been implicated.^9^ In contrast, experience of psychiatry during foundation training has been found to have a positive influence in choosing the specialty.^10,11^ During medical school, the length, quality of teaching and types of patient interaction have been identified as important.^12^

Knowledge of the push and pull factors for the specialty has led to the development of a number of initiatives to promote recruitment. University-based enrichment activities^13,14^ such as Psych Socs,^15^ special study modules (SSMs) and summer schools have emerged to support and encourage medical students with an interest in the field. Nationally, the Royal College of Psychiatrists (RCPsych) has developed the Psych Star Scheme and student Associate role, to enable enthusiastic medical students to further their interest in the field.^16^ Following graduation, ‘flexible pay premia’ encourage applications for hard-to-fill posts,^17^ including psychiatry. In parallel, the RCPsych's Choose Psychiatry campaign promotes psychiatry to medical students and foundation doctors through the use of videos, social media and podcasts.^18^

All of these complement the recent expansion in the number of psychiatry posts within the UK Foundation Programme. This was a direct response to concerns regarding recruitment into psychiatry and was recommended in the 2014 Broadening Foundation Programme report.^19^ It is recognised that the structure of the NHS and provision of healthcare need to change to meet the challenges of treating a population that is older, with increasing numbers of chronic health conditions. It emphasised the need for the Foundation Programme to provide broad-based training for junior doctors, giving them adequate experience and education in community and mental healthcare.^19^ Key for psychiatry was the recommendation that 22.5% of Foundation Year 1 (FY1) doctors and 22.5% Foundation Year 2 doctors (FY2) should have a post in psychiatry.^19^ Alongside the hope that this would boost recruitment into the specialty, it also represented an acknowledgement that there are numerous skills, including communication, empathetic understanding and multidisciplinary learning, acquired within a psychiatric placement, that are valuable whatever specialty the post holder ends up practising in.^20^

Despite this increase, many foundation doctors still have limited clinical experience of psychiatry. To address this the RCPsych, alongside the UK Foundation Programme, has developed Psychiatry Foundation Fellowships.^21^ These offer access to additional education and supervision resources to support doctors with an interest in the field during their foundation years through to specialty application. Academic Foundation Posts in psychiatry offer opportunities for trainees interested in research, directly challenging the negative assumptions regarding the lack of scientific basis to the field.^8^ Additionally, pilot Longitudinal Integrated Foundation Training (LIFT) posts in psychiatry are being developed, following the success of these in general practice.^22^

A recent announcement by the RCPsych suggests that these initiatives are working. In England, fill rates for core psychiatry training have increased for the third year running, with almost 100% of places filled for August 2020.^23^

## The impact of COVID-19 on medical education

On 12 March 2020, all doctors in the UK were informed that they would need to respond ‘rapidly and professionally’ to the challenge of COVID-19 and might be required to work in areas outside their usual practice.^24^ Although it was initially suggested that foundation doctors currently in mental health placements would not be redeployed,^2^ different local arrangements meant that some were moved from psychiatry to front-line roles.^25^ This was followed by the cancellation of all medical training rotations in April, May, June and July.^2,26^

There are approximately 15 000 foundation doctors in the UK,^27^ and thanks to the changes to the Foundation Programme, a significant number now undertake a post in psychiatry across their 2-year foundation training. One-third of these were due to start on 1 April 2020 but did not. If undertaking a high-quality psychiatry post during foundation training increases the likelihood that a trainee will apply to the specialty,^20^ this is likely to have a negative impact on the recruitment cycles for 2021 and 2022.

Undergraduate medical education has also faced significant upheaval. In the early weeks of March 2020, universities across the UK suspended face-to-face teaching and other educational activities. For medical students this meant the widespread cancellation of their clinical and elective placements,^3^ and a move to online education. While final year medical students were being fast-tracked through pre-registration to support the front line,^28^ students in other years missed out on vital educational experiences.^29^ The impact of these changes is already emerging.^30^ A recent survey of final year medical students found that over a third had their final year objective structured clinical examinations (OSCEs) cancelled and almost half had assistantships cancelled, with significant effects on self-reported ratings of preparedness to start as FY1 doctors.^30^

For psychiatry recruitment, the effects could be profound. Psychiatry placements often take place in the penultimate year of medical school. These students will now move into their final year, where the focus is on preparing for practice, with little time in the curriculum to make up for missed clinical placements. Given the uncertainty regarding the status of clinical placements during a second wave of the pandemic, it is highly likely that further year groups will similarly miss out on vital face-to-face psychiatry experience. In addition, the impact of the cancellation of the SSMs, elective placements and other enrichment activities, known to be key to fostering positive attitudes to a career in the specialty,^13–15^ is perhaps more concerning. These activities are available throughout medical school. The legacy of these losses therefore, means that the after-effects of the pandemic on psychiatric recruitment could be felt for years.

## The impact of COVID-19 on mental health

The potential impact on recruitment is particularly concerning given the postulated effects of the COVID-19 pandemic on the population's mental health.^31^ There is already evidence of psychological sequelae in those who were infected with SARS-Cov-2.^32^ More broadly, the impact of the public health measures, including shielding, social distancing and quarantine, is starting to emerge; a recent national survey demonstrated an increase in mental health problems across all age groups in April 2020.^33^ In the longer term, the consequences of the predicted economic recession^34^ will further increase psychiatric morbidity. These effects will be against a back-drop of a pre-pandemic predicted increase in mental healthcare needs within the population^35^ and ongoing workforce supply difficulties.^36^

## Opportunities to mitigate the potential impact of COVID-19 on recruitment into psychiatry

What can be done and what are the opportunities? Over the coming months, there will hopefully be some time to consider what we can do to mitigate any negative impact the acute response to COVID-19 has on recruitment into psychiatry. There are a number of dimensions to this.

Foundation trainee rotations have now resumed. In addition to the recommendations for these placements made by the RCPsych,^37^ trainers should take the opportunity to highlight some of the positive effects the NHS response to the pandemic has had on the delivery of mental healthcare, for example the cross-specialty collaborations demonstrated by initiatives such as the CoroNerve,^38^ the potential for telehealth, or new research avenues such as the role of the immune system in emerging psychiatric symptoms.^32^ These clearly demonstrate how central psychiatry is to the health and scientific communities' response to COVID-19, helping challenge many of the negative views of the specialty.

For those doctors who missed out on their chance to undertake a psychiatry foundation post, there are other opportunities for their potential enthusiasm to be encouraged. Existing initiatives for foundation doctors should be strengthened,^39^ with targeted invites to those doctors affected by the cancellation of rotations, if possible. For FY1 doctors there remains the opportunity to undertake taster days in psychiatry as FY2s. These are usually limited, but given the flexibility foundation doctors demonstrated to facilitate the NHS's acute COVID-19 response, supporting them to make up for lost clinical experience by extending these seems reasonable.

Foundation doctors are increasingly not applying directly to specialty training, instead undertaking F3 and F4 years.^6^ The expansion of clinical fellowships in psychiatry – which offer full access to supervision and the other educational opportunities that core trainees receive – may offer alternative opportunities for those who missed out first time round.^40^

The Medical Schools Council has outlined the need for clinical placements to restart,^41^ with priority given to those closer to graduation. Further cohorts of medical students are therefore likely to be affected by the loss of clinical experience in psychiatry. In recent years, virtual work experience programmes have been developed to support school-age students considering a career in medicine.^42^ Universities should consider learning lessons from these, to create virtual psychiatry placements, with an emphasis on patient interaction and high-quality clinical teaching. This could complement the number of psychiatric summer and autumn schools that have already moved online.^43^

Although developing imaginative alternatives to face-to-face clinical placements is important, it is essential that this does not become the default. Positive attributes of undergraduate psychiatry education include time on placement, working directly with the multidisciplinary team and the influence of role models from within the psychiatric team.^44^ It is hard to see how these can be achieved remotely. The continued facilitation of face-to-face psychiatry experience for medical students should therefore remain a priority.

It has been suggested that time for enrichment activities such as SSMs should be redirected towards core clinical placement activity.^41^ It is vital that undergraduate psychiatry departments are involved in these discussions. Not only are enrichment activities in psychiatry important for recruitment, they help fight stigma towards the specialty and the patient population. These should be viewed not as optional extras, but as essential to creating a generation of doctors who view mental illness in parity with physical illness.

One of the positive outcomes of the COVID-19 pandemic has been the speed and readiness with which medical education departments have adapted to the use of online learning. The Medical Schools Council is recommending the use of a number of online platforms to support undergraduate education.^45^ It is vital that psychiatric education is embedded throughout these. The potential for moving SSMs and other psychiatric enrichment activities online should also be fully explored. The use of webinars has greatly expanded as a result of the pandemic, with the RCPsych producing a number that are freely available, and grand rounds and journal clubs now frequently take this form. Delivering psychiatric education this way offers great scope to widen the potential audience, with the main barrier being the lack of awareness among potential attendees. At a national level these resources should be highlighted within the Choose Psychiatry campaign. More locally, promotion of online educational material should be embedded within induction for medical students and via local postgraduate education departments.

The expansion of simulation for psychiatry is another area of potential.^46^ These courses should be re-offered to those who missed out on their clinical placements, as well as expanded to supplement loss of clinical experience of future students. Although much postgraduate education remains online, face-to-face foundation simulation programmes continue. A number of providers have already developed psychiatric simulation courses for foundation doctors, but frequently these are available only to trainees undertaking a psychiatry post. The skills of managing risk, communication, human factors and de-escalation, inherent in these courses, are key outcomes of the Foundation Programme Curriculum^47^ and could easily be incorporated into existing programmes. Through their inclusion alongside other ‘core’ subjects, psychiatric simulation courses would reduce stigma as well as encourage some to consider a future career in the specialty.

## Conclusions

Developing a set of strengthened undergraduate and postgraduate psychiatric educational resources can only benefit future recruitment into the specialty. But as autumn continues, we are now are facing the second wave of COVID-19 cases. Although the redeployments of healthcare workers and cancellations of placements were necessary first time round, it is essential that we reflect on their impact. Understanding the effects of the acute management of the COVID-19 pandemic on psychiatric recruitment is vital to inform decisions regarding future suspensions of medical student attachments and movements of trainee doctors. These must try to balance the acute requirements of COVID-19 patients with the need to ensure that there is an adequate psychiatric workforce to address not only the current, but also future mental health repercussions of the pandemic.

## About the author

Flora Greig is a general adult and old age psychiatry registrar at South London and Maudsley NHS Foundation Trust, Croydon University Hospital, London, UK.

## Supplementary material

For supplementary material accompanying this paper visit https://doi.org/10.1192/bjb.2020.112. click here to view supplementary material
